# Impact of Frailty on Inpatient Mortality and Resource Utilization for Primary Pulmonary Hypertension

**DOI:** 10.1055/s-0044-1801349

**Published:** 2025-01-08

**Authors:** Rayaan Rauf, Cheryl A. Gibson, Mohamad Alhoda Mohamad Alahmad

**Affiliations:** 1 School of Medicine, University of Missouri–Kansas City, Kansas City, Missouri, United States; 2 Department of Internal Medicine, University of Kansas Medical Center, Kansas City, Kansas, United States

**Keywords:** frailty, primary pulmonary hypertension, inpatient mortality

## Abstract

**Background** Frailty has been associated with inferior outcomes in patients with primary pulmonary hypertension (PPH). There is a lack of national data to assess if hospital frailty risk score (HFRS) is associated with worse inpatient outcomes in PPH.

**Methods** Our retrospective study used the Nationwide Readmission Database (NRD). First, we extracted all cases older than 18 years who were discharged with a principal diagnosis of PPH between January and November 2016 to 2019 to allow for a 30-day follow-up. Appropriate survey and domain analyses were applied to obtain national estimates using SAS 9.4.

**Results** We identified 4,555 cases. HFRS <5 was present in 56% ( *n* = 2,555) of the cohort. Patients with an intermediate-to-high frailty risk score (HFRS ≥5) were older than those with a low frailty risk score (HFRS <5), with a mean age of 61 versus 54 years ( *p* < 0.01), and had slightly fewer women (75 vs. 78%, *p* = 0.09). Patients with HFRS >5 had a higher prevalence of dementia, depression, diabetes mellitus, malignancy, acute encephalopathy, coagulopathy, heart failure, and chronic (liver and renal) diseases ( *p* < 0.01). Also, they had higher inpatient mortality during index admission (14 vs. 2%, *p* < 0.001), and all-cause 30-day readmission rates (38 vs. 33%, *p* = 0.01). Univariate analysis suggests a positive correlation between the degree of frailty and the odds of inpatient mortality (referenced to HFRS <5). The HFRS 5 to 10 group has an odds ratio (OR) of 5 (95% confidence interval [CI]: 3.3–8), the HFRS 10 to 15 group has an OR of 14 (95% CI: 8–23), and the HFRS >15 group has an OR of 20 (95% CI: 9–45). Even after adjusting for age, gender, and significant comorbidities, the single most important factor associated with higher odds of inpatient mortality was HFRS >5 (OR: 5.5 [95% CI: 3.7–8.3], *p* < 0.001) followed by acute myocardial infarction, acute encephalopathy, heart failure, chronic liver disease, and malnutrition. Length of stay had linear trend with HFRS (mean of 6 days for HFRS <5 vs. 11 days for HFRS 5–10 vs. 19 days for HFRS >10, *p* < 0.001).

**Conclusion** Adverse inpatient outcomes correlate with the severity of HFRS in PPH.

## Introduction

Primary pulmonary hypertension (PPH), a rare and progressive condition marked by elevated blood pressure in pulmonary arteries, leads to significant morbidity and mortality. 1 Inactivating mutations in the *BMPR2* gene are the most common genetic cause of PPH, leading to dysregulation of pulmonary vascular remodeling. 2

Frailty, a clinical syndrome characterized by decreased reserve and resistance to stressors, is assessed by the hospital frailty risk score (HFRS), a tool designed to quantify the risk of frailty in hospitalized patients based on the International Classification of Diseases, Tenth Revision (ICD-10) codes, which helps in identifying patients at higher risk of poor outcomes. 3 4 5 6 Despite the significance of frailty in PPH outcomes, there is a notable lack of comprehensive data evaluating the influence of HFRS on inpatient outcomes among PPH patients.

The association between frailty and adverse outcomes in PPH patients can be attributed to various factors. 7 8 9 Frailty is a complex syndrome involving decreased physiological reserve and resistance to stressors, and in the context of PPH, it may reflect the cumulative impact of cardiovascular and respiratory impairments, as well as the burden of comorbidities. 10 11 12 13 Additionally, frailty is often accompanied by systemic inflammation, hormone resistance, and increased muscle protein degradation, which further exacerbate the vulnerability of PPH patients to adverse events. 4 14 15

## Methods

ICD-10 is a system to code all medical diagnoses, symptoms, and procedures designed by the World Health Organization. The system is modified by Centers for Medicare and Medicaid Services and the National Center for Health Statistics to better fit the U.S. health system. 6 Hospital billing data include patient demographics and ICD-10 codes. The Agency for Healthcare Research and Quality (AHRQ) is a federal agency under the Department of Health and Human Services that standardizes states billing data obtained from different hospitals in each state to create uniform databases such as Nationwide Readmission Database (NRD). NRD is a publicly available database designed to generate national estimates of all-cause and condition-specific inpatient readmissions. Quality measures are applied to ensure accuracy. The study was deemed exempt by the Institutional Review Board as the NRD contains deidentifying patients' information.

**Fig. 1:**
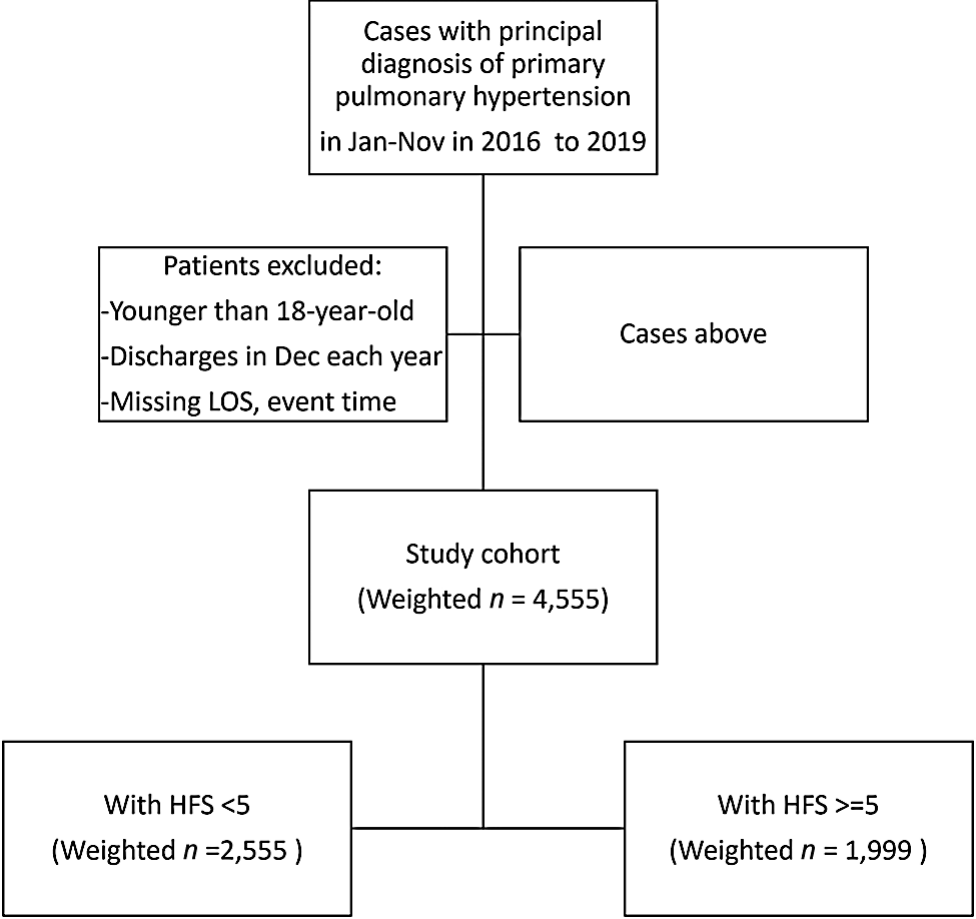
Study design. HFRS, hospital frailty risk score; LOS, length of stay.

For this study, we used NRDs from 2016 as we are interested in utilizing ICD-10 codes only. We did not include data after 2019 to avoid possible bias related to the coronavirus disease 2019 pandemic. We included all patients aged 18 years and older with a primary diagnosis (I10_Dx1) of PPH who were admitted from January to November in each year studied. We calculated HFRS for each case. HFRS is an ICD-10-based score validated to provide a low-cost systematic way to screen for frailty and predict hospital adverse outcomes. 14 To simplify the analysis, we characterized patients into two categories based on HFRS: low frailty score (HFRS <5) and high frailty score (HFRS ≥5), as shown in Fig. 1 . The latter can be categorized into three classes: HFRS 5 to 10, HFRS 10 to 15, and HFRS above 15, as shown in Supplementary Table S1 (online only).

**Table 1:** Baseline characteristics

Variable	Total	With HFRS >5	With HFRS <5	*p* -Value
Age, mean (SD)	56.8 (23.2)	60.6 (21.3)	53.8 (23.9)	<0.0001
Female gender	3,498 (76.8%)	1,499 (75.0%)	1,999 (78.2%)	0.0936
Chronic lung disease	1,629 (35.8%)	809 (40.5%)	820 (32.1%)	<0.0001
Dementia	65 (1.4%)	52 (2.6%)	13 (0.5%)	<0.0001
Depression	684 (15.0%)	352 (17.6%)	332 (13.0%)	0.0019
Diabetes mellitus	1,301 (28.6%)	693 (34.7%)	608 (23.8%)	<0.0001
Hypertension	1,940 (42.6%)	1,039 (52.0%)	901 (35.3%)	<0.0001
CHF	2,995 (65.8%)	1,569 (78.5%)	1,426 (55.8%)	<0.0001
Malignancy	187 (4.1%)	125 (6.3%)	62 (2.4%)	<0.0001
Obesity	1,221 (26.8%)	579 (29.0%)	642 (25.1%)	0.032
PVD	238 (5.2%)	135 (6.8%)	102 (4.0%)	0.0016
Deficiency anemia	1,029 (22.6%)	593 (29.7%)	435 (17.0%)	<0.0001
Blood loss	36 (0.8%)	22 (1.1%)	13 (0.5%)	0.0764
Coagulopathy	814 (17.9%)	511 (25.6%)	303 (11.9%)	<0.0001
Chronic liver disease	633 (13.9%)	364 (18.2%)	269 (10.5%)	<0.0001
Movement disorder	101 (2.2%)	58 (2.9%)	43 (1.7%)	0.0646
Seizure	122 (2.7%)	84 (4.2%)	38 (1.5%)	0.0006
Encephalopathies	143 (3.1%)	123 (6.2%)	20 (0.8%)	<0.0001
Paralysis	48 (1.0%)	38 (1.9%)	9 (0.4%)	0.0009
Psychosis	161 (3.5%)	87 (4.4%)	73 (2.9%)	0.0794
CKD	1,102 (24.2%)	806 (40.3%)	296 (11.6%)	<0.0001
PUD	31 (0.7%)	16 (0.8%)	15 (0.6%)	0.5265
CBVD (POA)	58 (1.3%)	39 (2.0%)	19 (0.7%)	0.0026
CBVD sequelae	31 (0.7%)	28 (1.4%)	3 (0.1%)	<0.0001
Hospital location				
Central metropolitan	1,219 (26.8%)	520 (26.0%)	698 (27.3%)	0.0647
Fringe metropolitan	1,217 (26.7%)	596 (29.8%)	621 (24.3%)	
Medium metropolitan	831 (18.3%)	330 (16.5%)	501 (19.6%)	
Small metropolitan	514 (11.3%)	226 (11.3%)	289 (11.3%)	
Micropolitan counties	435 (9.5%)	190 (9.5%)	245 (9.6%)	
Other	321 (7.0%)	129 (6.5%)	192 (7.5%)	
Socioeconomic status				
1	1,223 (26.9%)	505 (25.3%)	718 (28.1%)	0.1294
2	1,184 (26.0%)	505 (25.3%)	678 (26.6%)	
3	1,142 (25.1%)	510 (25.5%)	632 (24.7%)	
4	958 (21.0%)	457 (22.9%)	501 (19.6%)	

Abbreviations: CBVD, cerebrovascular disease; CHF, chronic heart failure; CKD, chronic kidney disease; HFRS, hospital frailty risk score; POA, present on admission; PUD, peptic ulcer disease; PVD, peripheral vascular disease; SD, standard deviation.

We examined comorbidities that are related to the risk of inpatient mortality and readmission, which are included in the AHRQ Elixhauser Comorbidity Score and have been validated to predict both inpatient mortality and 30-day readmissions. 16 To maintain privacy, ethnicity is not available in NRD datasets.

Complex survey design, weights, and clustering were considered during the analysis using “Survey” procedures in Statistical Analysis Software (SAS), producing a nationwide analysis for discharge estimates from almost all hospitals in the United States. Categorical and continuous variables were reported as percentages and mean ± standard deviation (SD), respectively. Differences in mean and percentage were assessed using least-squares means and chi-squared tests, respectively. Logistic regression was used to analyze the independent impact of frailty on categorical outcomes. The final parsimonious model included age, gender, encephalopathy, chronic heart failure, chronic liver disease, and frailty. Statistical significance was considered as *p* -value < 0.05. All analyses were performed using SAS version 9.4 (SAS Institute Inc., SAS 9.4, Cary, North Carolina, United States).

## Results

We classified patients included with PPH into two categories to simplify the study: those at low risk for frailty (HFRS < 5) and those likely to have frailty (HFRS ≥ 5). Almost half of the cases (44%) were found to be at risk for frailty (HFRS > 5).

Table 1 shows that patients at risk for frailty were older, with a mean age of 60 years (compared with 53 years for patients at low risk for frailty, *p* -value 0.0001) and had a similar distribution of female sex. The majority of patients were female (77%).

Patients at risk for frailty were significantly ( *p* -value < 0.05) associated with a higher frequency of chronic comorbidities, including chronic lung disease, chronic kidney disease, chronic liver disease, chronic heart failure, hypertension, diabetes mellitus, obesity, acute encephalopathies, dementia, depression, cerebrovascular disease, malignancies, and malnutrition.

**Fig. 2:**
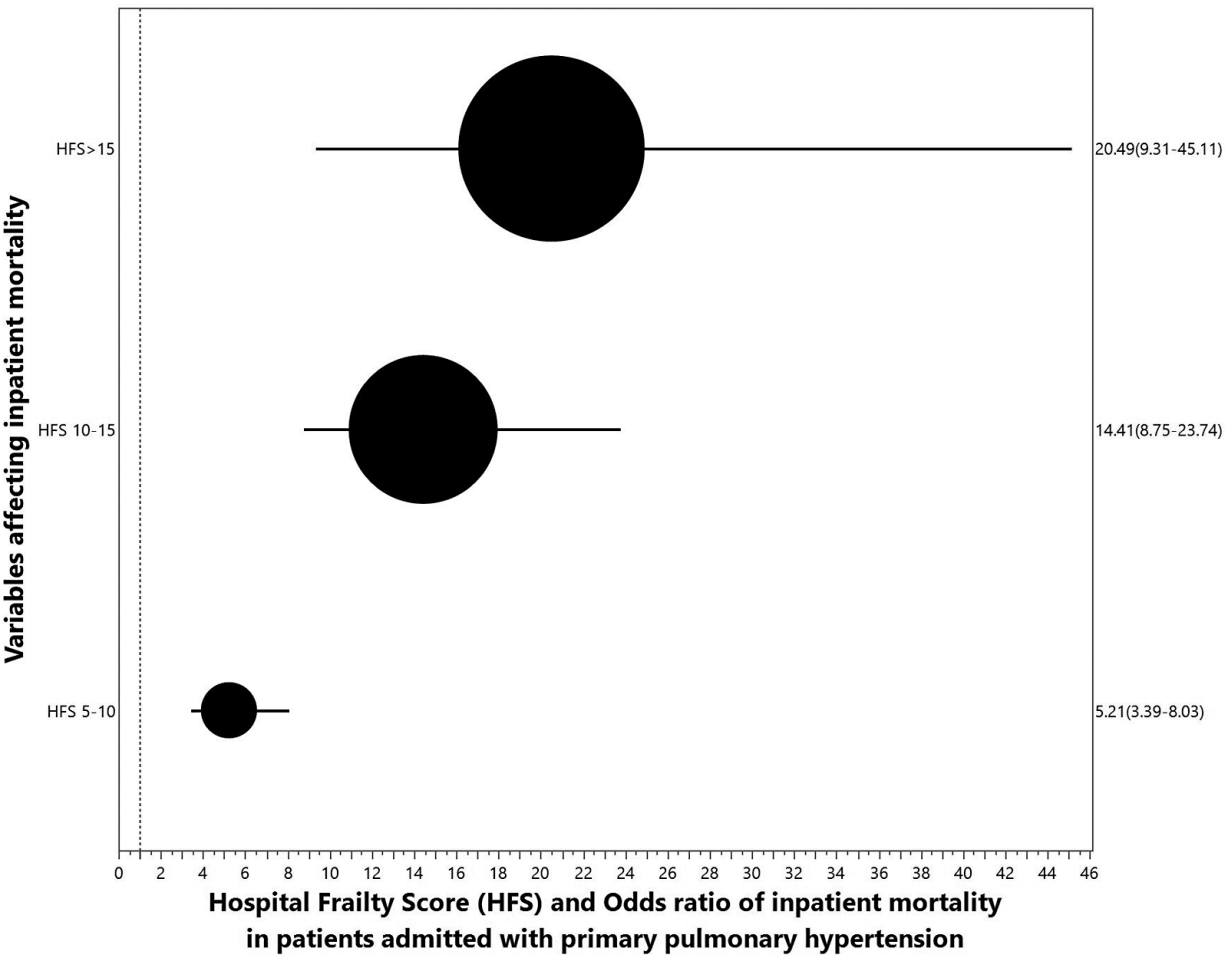
HFRS and mortality. HFRS, hospital frailty risk score.

**Fig. 3:**
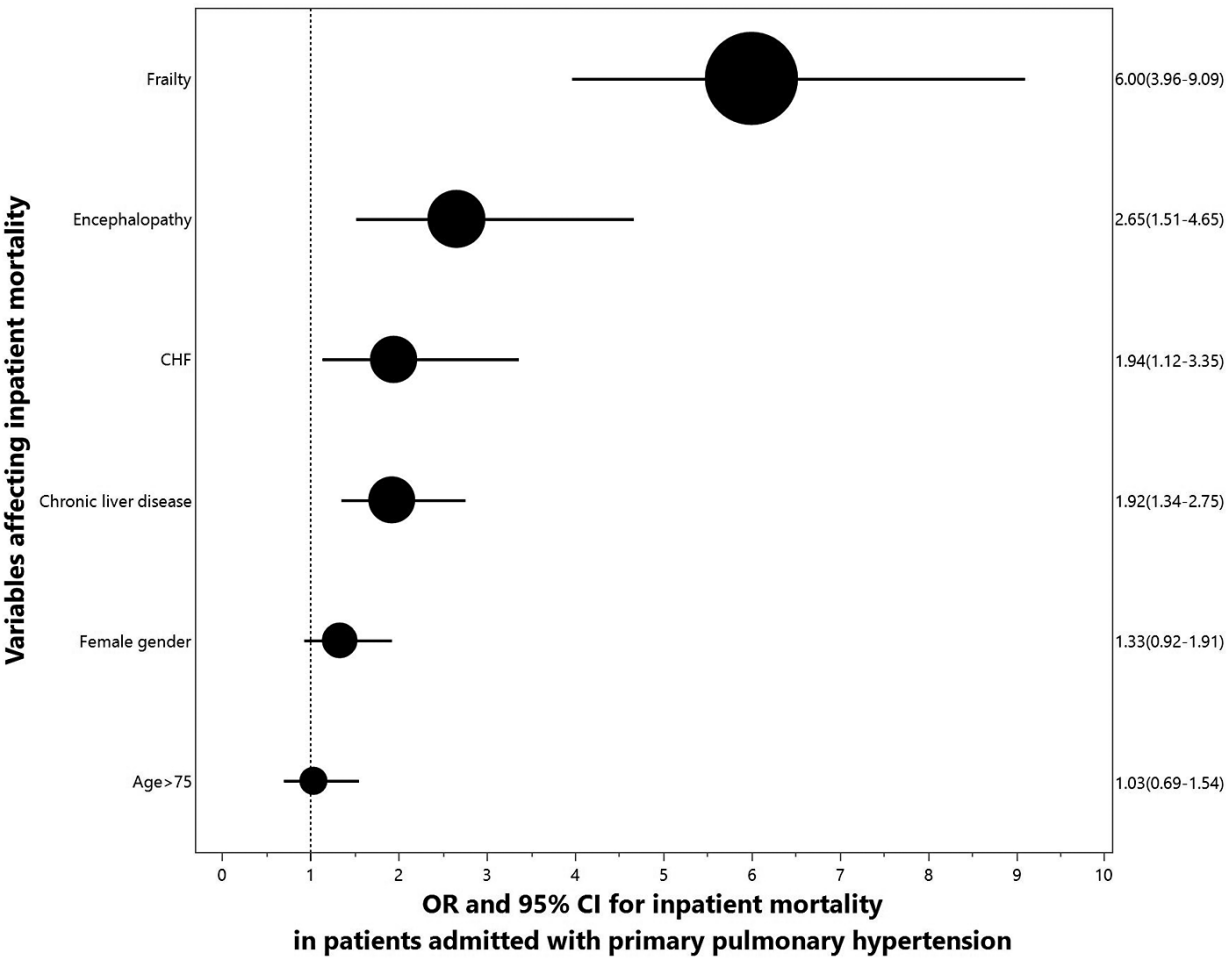
Multivariate analysis. CI, confidence interval; OR, odds ratio.

Univariate analysis showed that the odds ratio for inpatient mortality is 7.6 times higher in patients who are frail (5.1–11.3). The higher the score, the higher the odds for inpatient mortality ( Fig. 2 ). Multivariate analysis ( Fig. 3 ) showed that even after adjustment for significant comorbidities, age, and gender, HFRSs more than 5 were the most important single factor associated with higher inpatient mortality (adjusted odds ratio: 6 [95% CI: 4–9], *p* -value 0.0001). Table 2 shows that cases at risk for frailty (HFRS >5) also had a prolonged hospital stay (13 vs. 6 days, *p* -value < 0.0001) and subsequently total hospitalization charges ($212,609 vs. $77,775, *p* -value < 0.001). They also had a higher rate of 30-day daily admission (19 vs. 13%, *p* -value < 0.001) and a slight increase in inpatient mortality during first readmission after the index hospitalization (12 vs. 8%, *p* -value 0.095).

**Table 2:** Outcomes

Variable	Total	With HFRS >5	With HFRS <5	*p* -Value
Index mortality	329 (7.2%)	277 (13.9%)	52 (2.0%)	<.0001
Index malnutrition	459 (10.1%)	337 (16.9%)	122 (4.8%)	<0.0001
Index LOS, mean (SD)	9.0 (16.8)	13.3 (21.7)	5.6 (9.5)	<0.0001
Total charges mean (SD)	136,936 (516,428)	212,609 (680,305)	77,775 (311,036)	<0.0001
30-d readmission	692 (15.2%)	372 (18.6%)	320 (12.5%)	<0.0001
Readmission mortality	70 (10%)	45 (12%)	25 (8%)	0.0953

Abbreviations: HFRS, hospital frailty risk score; LOS, length od stay; SD, standard deviation.

## Discussion

This study aimed to investigate the association between frailty, as measured by the HFRS, and inpatient outcomes among patients diagnosed with PPH at a national level.

The HFRS has emerged as a valuable tool for identifying frailty in hospitalized patients using routinely collected administrative data to quantify frailty and predict adverse outcomes such as prolonged hospital stays, readmissions, and mortality in a large cohort of older adults. 10 14

In examining the impact of frailty on increased mortality and disease progression, our findings align with and expand upon the existing literature. Boyd et al highlighted that frailty significantly increased hospitalization rates and disability progression among older women, suggesting a similar trajectory of adverse outcomes in frail populations, which parallels our findings in PPH patients, where higher frailty scores were associated with increased inpatient mortality and prolonged hospital stays. 5 Wang et al and Guan and Niu further reinforced the notion that frailty exacerbates disease progression and adverse events in chronic respiratory conditions, supporting our results that frailty significantly worsens the prognosis for PPH patients. 12 17 Furthermore, Dinesh et al explored the relationship between rehabilitation and frailty in patients with advanced heart or lung disease, demonstrating that frailty is a significant predictor of poor surgical outcomes, underscoring the necessity for targeted rehabilitation strategies to mitigate these risks. 18

Resource utilization in the hospital setting is markedly influenced by frailty, as demonstrated in our study and corroborated by the existing literature. Bernabeu-Mora et al showed that frailty was a predictive factor for 90-day readmissions following hospitalization for chronic obstructive pulmonary disease exacerbations, a finding that aligns with our observation of higher readmission rates among frail PPH patients. 4 Hadaya et al examined the impact of frailty on clinical outcomes and resource use following emergency general surgery, reporting increased hospital costs, longer stays, and higher readmission rates for frail patients, which parallels our findings of significantly higher hospitalization charges and longer stays for frail PPH patients. 9

Our findings are consistent with other studies, such as Makary et al, which showed that frailty predicts increased health care resource use, including longer hospital stays and higher costs. 8

Recent studies in older populations indicate that frailty may be reversible with targeted exercise and nutritional interventions, although evidence supporting nutritional strategies remains limited. 19 20 Overall, these studies collectively highlight the substantial health care burden imposed by frailty, reinforcing the necessity for tailored management strategies to mitigate resource utilization and improve outcomes in frail PPH patients.

The strengths of our study include the use of a large national database, which allowed for a comprehensive analysis and broad applicability of our findings. However, the study also has several limitations. First, the retrospective nature of the study limits our ability to establish causality between frailty and inpatient outcomes. Additionally, the lack of detailed clinical data (e.g., laboratory results and imaging) may have influenced our findings. Another limitation of the study is that the HFRS was not developed for the NRD (or any other U.S. database), which may underestimate frailty due to limited outpatient data integration. The score also was developed for patients above the age of 75 years, raising questions about its reliability in younger populations, such as those with PPH. Additionally, PPH patients often undergo comprehensive evaluations, which may reduce the utility of the HFRS in this context. Prospective studies are needed to validate our findings, establish causative relationships, and explore the efficacy of various interventions, including nutritional support and exercise programs, in improving outcomes for all frail PPH patients.

## Conclusion

In conclusion, our study demonstrates that frailty assessment, using HFRS, can be easily incorporated into clinical practice. It is a significant predictor of adverse inpatient outcomes in patients with PPH. Frailty is associated with higher hospital mortality, increased 30-day readmission rates, and prolonged hospital stays. Further research is necessary to confirm our findings and to develop and test interventions aimed at reducing the impact of frailty in PPH patients.
