# Supplementary material for: Impact of Frailty on Inpatient Mortality and Resource Utilization for Primary Pulmonary Hypertension
Source: Avicenna J Med. 2025 Jan 8;14(4):204–9. doi: 10.1055/s-0044-1801349 (PMC11896733; doi:10.1055/s-0044-1801349)
Supplement: Supplementary file 1 — Supplementary Material [file 10-1055-s-0044-1801349-s240135.pdf]

Supplementary Table S1 Distribution of frailty based on hospital frailty risk score (HFRS).

Variable	Total
Frailty	
HFRS <5	2,555 (56.1%)
HFRS 5–10	1,475 (32.4%)
HFRS 10–15	413 (9.1%)
HFRS >15	112 (2.5%)
Total	4,555 (100.0%)

Abbreviation: HFRS, hospital frailty risk score.

 © 2025. The Author(s).
